# Supplementary material for: Upper Cervical Manipulation and Manual Massage Do Not Modulate Sympatho-Vagal Balance or Blood Pressure in Women: A Randomized, Placebo-Controlled Clinical Trial
Source: Healthcare (Basel). 2025 Oct 10;13(20):2554. doi: 10.3390/healthcare13202554 (PMC12562330; doi:10.3390/healthcare13202554)
Supplement: Supplementary file 1 [file healthcare-13-02554-s001.zip › healthcare-3851900-supplementary/Data Collection.pdf]

**Subjects**	**Dominant Lower Limb**	**Age (years)**	**Weigth (kg)**
1	Right	21	57,3
2	Left	19	56,4
3	Left	18	54,9
4	Right	21	53,8
5	Right	20	50,7
6	Right	34	65,3
7	Right	18	50,2
8	Right	20	81,3
9	Right	20	66,2
10	Right	32	64,2
11	Right	28	58,2
12	Right	29	57,4
13	Right	26	53,4
14	Right	18	50,7
15	Righ	22	66,6
Means		23,07	59,11
Sd		5,34	8,35

Height (m)	BMI	Physical Activity Level
1,67	20,55	270
1,64	20,97	185
1,58	21,99	750
1,66	19,52	580
1,71	17,34	290
1,69	22,86	1340
1,63	18,89	720
1,64	30,23	255
1,72	22,38	210
1,65	23,58	1100
1,62	22,18	1850
1,73	19,18	250
1,57	21,66	920
1,49	22,84	150
1,66	24,17	135
1,64	21,89	600,33
0,06	2,99	513,35

Systolic Blood Pressure mmHg						
**Suibjects**	**Baseline**	**Post-0**	**Post-15**	**Post-30**	**Post-45**	**Post-60**
1	111	115	109	113	108	117
2	112	112	116	123	116	118
3	116	120	118	120	120	116
4	102	91	95	99	108	97
5	107	104	102	104	107	107
6	122	113	112	111	111	118
7	115	108	106	105	108	105
8	115	99	103	100	100	109
9	107	105	107	100	107	101
10	93	103	102	94	107	112
11	110	114	112	112	114	114
12	103	111	101	96	100	108
13	103	109	110	108	103	115
14	102	97	94	105	98	107
15	100	97	104	100	100	96
**Means**	107,87	106,53	106,07	106,00	107,13	109,33
**Sd**	7,56	8,06	6,95	8,48	6,33	7,30
**Median**	107,00	108,00	106,00	105,00	107,00	109,00

Diastolic Blood Pressure mmHg					
**Suibjects**	**Baseline**	**Post-0**	**Post-15**	**Post-30**	**Post-45**
1	72	74	71	73	76
2	74	67	75	77	86
3	82	80	80	78	80
4	67	64	61	72	68
5	59	62	63	63	74
6	78	72	75	75	95
7	75	73	68	72	80
8	79	77	81	77	82
9	59	62	67	62	63
10	62	67	70	66	70
11	78	80	80	76	80
12	69	73	72	74	74
13	68	68	70	64	74
14	66	70	63	62	66
15	70	71	70	72	72
**Means**	70,53	70,67	71,07	70,87	76,00
**Sd**	7,22	5,78	6,27	5,84	8,20
**Median**	70,00	71,00	70,00	72,00	74,00

Post-60
77
76
78
77
68
76
78
80
65
75
78
72
74
73
71
74,53
4,12
76,00

		RMSSD ms		
Suibjects	Baseline	Post-0	Post-15	Post-30
1	29.34	48.75	48.75	40.71
2	71.58	87.62	68.14	58.63
3	105.13	110.70	115.15	107.15
4	49.83	74.25	82.42	73.92
5	17.98	27.71	39.99	47.80
6	32.86	51.49	45.68	47.96
7	50.95	95.60	98.24	84.92
8	102.42	58.01	65.97	49.30
9	40.05	47.37	43.15	30.92
10	31.45	27.00	61.52	61.18
11	40.75	63.39	55.53	46.83
12	25.90	107.57	52.21	33.38
13	16.83	30.18	65.66	64.21
14	28.08	70.25	72.05	65.29
15	79.59	109.70	34.99	31.80
**Means**	#DIV/0!	#DIV/0!	#DIV/0!	#DIV/0!
**Sd**	#DIV/0!	#DIV/0!	#DIV/0!	#DIV/0!
**Median**	#NUM!	#NUM!	#NUM!	#NUM!

SDNN ms

Suibjects	Baseline	Post-0	Post-15	Post-45	Post-60
1	41.08	48.16	46.34	44.62	56.88
2	79.88	73.06	92.05	74.44	62.64
3	94.08	113.06	114.57	102.18	98.51
4	64.72	84.24	82.81	72.59	79.40
5	25.30	34.19	71.74	48.66	44.42
6	48.45	58.65	96.73	42.15	52.23
7	52.84	74.55	79.16	82.45	75.92
8	93.09	73.50	85.72	63.49	49.37
9	56.52	46.98	48.62	44.04	16.10
10	38.73	127.00	87.73	78.88	85.31
11	59.44	54.97	63.02	19.41	25.95
12	45.32	116.36	50.22	35.05	49.99
13	44.20	38.03	95.78	90.52	33.91
14	44.44	73.78	70.53	76.12	58.43
15	130.15	105.92	69.79	35.71	39.98
**Means**	#DIV/0!	#DIV/0!	#DIV/0!	#DIV/0!	#DIV/0!
**Sd**	#DIV/0!	#DIV/0!	#DIV/0!	#DIV/0!	#DIV/0!
**Median**	#NUM!	#NUM!	#NUM!	#NUM!	#NUM!

**Post-30**	**Post-45**	**Post-60**
58.63	44.50	93.19
113.87	92.71	91.49
103.72	105.89	101.79
84.52	86.16	86.96
83.07	75.61	79.84
91.57	96.62	81.39
91.51	76.10	62.02
61.79	87.96	52.30
48.00	59.50	52.87
61.72	85.72	97.47
74.01	62.08	71.88
45.04	62.51	68.31
86.04	87.71	78.51
78.12	78.85	77.36
64.96	77.10	79.01
#DIV/0!	#DIV/0!	#DIV/0!
#DIV/0!	#DIV/0!	#DIV/0!
#NUM!	#NUM!	#NUM!

		**LN (**
**Suibjects**	**Baseline**	**Post-0**
1	3.38	3.89
2	4.27	4.47
3	4.66	4.71
4	3.91	4.31
5	2.89	3.32
6	3.49	3.94
7	3.93	4.56
8	4.63	4.06
9	3.69	3.86
10	3.45	4.84
11	3.73	2.98
12	3.25	4.68
13	2.82	3.41
14	3.34	4.25
15	4.38	4.70
**Means**	#DIV/0!	#DIV/0!
**Sd**	#DIV/0!	#DIV/0!
**Median**	#NUM!	#NUM!

RMSSD) ms			
**Post-15**	**Post-30**	**Post-45**	**Post-60**
3.84	3.71	3.80	4.04
4.22	4.07	4.31	4.14
4.75	4.67	4.63	4.59
4.41	4.30	4.28	4.37
3.69	3.87	3.88	3.79
3.82	3.87	3.74	3.96
4.59	4.44	4.41	4.33
4.19	3.90	4.15	3.90
3.76	3.43	3.79	2.78
4.12	4.11	4.37	4.43
3.22	3.85	2.97	3.26
3.96	3.51	3.56	3.91
4.18	4.16	4.51	3.52
4.28	4.18	4.33	4.07
3.55	3.46	3.58	3.69
#DIV/0!	#DIV/0!	#DIV/0!	#DIV/0!
#DIV/0!	#DIV/0!	#DIV/0!	#DIV/0!
#NUM!	#NUM!	#NUM!	#NUM!

**Suibjects**	**Baseline**
1	8
2	46
3	67
4	24
5	1
6	9
7	37
8	64
9	17
10	12
11	14
12	4
13	1
14	9
15	45
**Means**	23,87
**Sd**	22,36
**Median**	14,00

PNN50%						
**Post-0**	**Post-15**	**Post-30**	**Post-45**	**Post-60**		**Suibjects**
28	23	18	21	28		1
65	46	37	49	39		2
60	62	59	56	55		3
46	55	46	44	50		4
2	23	13	13	6		5
27	22	22	24	33		6
67	66	54	53	36		7
44	39	30	37	9		8
26	25	9	25	2		9
77	33	43	48	50		10
1	4	30	2	7		11
72	32	12	13	23		12
7	35	40	57	13		13
52	58	51	53	29		14
67	14	9	15	21		15
42,73	35,80	31,53	34,00	26,73		**Means**
26,08	18,36	17,11	18,67	17,21		**Sd**
46,00	33,00	30,00	37,00	28,00		**Median**

Mean R-R ms					
**Baseline**	**Post-0**	**Post-15**	**Post-30**	**Post-45**	**Post-60**
740.63	865.34	891.24	894.73	907.44	871.86
873.27	931.04	883.73	844.58	917.58	899.73
796.12	789.88	833.26	830.34	804.50	797.72
800.60	852.40	869.30	881.19	875.01	930.07
745.46	824.65	853.28	816.59	812.08	814.10
747.08	832.28	785.25	819.25	721.87	805.60
836.53	932.25	945.60	852.14	918.14	936.86
813.00	935.09	897.00	880.06	926.73	833.73
832.28	825.01	821.63	779.03	831.15	607.00
841.86	789.88	933.54	976.79	984.26	977.29
634.55	695.40	692.40	866.99	692.66	711.53
760.27	1119.64	864.56	832.32	820.89	848.22
641.17	678.85	763.80	787.90	880.27	733.98
612.49	734.20	767.55	729.37	745.48	667.60
738.39	861.55	868.47	881.14	927.04	921.18
#DIV/0!	#DIV/0!	#DIV/0!	#DIV/0!	#DIV/0!	#DIV/0!
#DIV/0!	#DIV/0!	#DIV/0!	#DIV/0!	#DIV/0!	#DIV/0!
#NUM!	#NUM!	#NUM!	#NUM!	#NUM!	#NUM!

TOTAL POWER ms2						
**Suibjects**	**Baseline**	**Post-0**	**Post-15**	**Post-30**	**Post-45**	**Post-60**
1	756.05	1794.55	1037.16	884.53	1053.19	3829.44
2	2760.83	3375.20	4269.74	3581.26	4816.04	3087.47
3	7779.94	10515.18	9526.98	6584.01	78255.58	6066.56
4	2484.04	6412.44	5069.66	4929.37	4946.58	4179.46
5	634.16	453.23	1855.42	4719.12	3039.01	3107.56
6	1343.97	2464.36	1902.52	3302.12	2381.87	5060.14
7	1479.96	5054.06	4108.85	4948.31	2696.38	3434.52
8	4541.55	2381.00	2471.27	2268.83	3537.17	1405.81
9	1346.67	1665.95	1272.61	938.59	1759.35	634.74
10	837.41	1129.65	3656.64	2101.27	3721.85	4254.52
11	2453.67	1015.29	1930.82	1772.19	1031.91	839.43
12	576.61	6552.77	1783.52	1505.62	2168.33	1705.97
13	356.45	893.91	4704.60	4429.94	6206.02	1426.43
14	969.63	4091.58	3923.70	3538.61	5319.73	3517.22
15	8371.30	5585.12	1670.29	1737.25	2148.39	2517.31
**Means**	#DIV/0!	#DIV/0!	#DIV/0!	#DIV/0!	#DIV/0!	#DIV/0!
**Sd**	#DIV/0!	#DIV/0!	#DIV/0!	#DIV/0!	#DIV/0!	#DIV/0!
**Median**	#NUM!	#NUM!	#NUM!	#NUM!	#NUM!	#NUM!

LF POWER ms2					
**Suibjects**	**Baseline**	**Post-0**	**Post-15**	**Post-30**	**Post-45**
1	334.20	734.90	409.38	410.20	407.76
2	1668.27	1467.53	3016.02	2301.86	2671.68
3	1626.79	5422.42	4166.09	3234.42	3349.68
4	1279.19	3651.38	1778.85	2556.42	2693.31
5	430.05	228.26	1113.66	3497.24	2032.06
6	650.67	1573.27	1244.11	2458.54	1896.11
7	464.22	1523.17	1131.69	2001.51	962.35
8	2034.44	1229.79	1540.16	1094.41	1795.87
9	687.76	831.11	517.32	556.79	787.63
10	391.74	4238.43	2251.72	1117.90	1951.40
11	1629.27	868.31	1693.83	915.33	891.20
12	256.48	3489.56	668.92	1139.53	1682.80
13	274.20	412.50	2912.51	2370.40	2401.02
14	480.93	1039.67	787.29	909.31	1582.67
15	5183.12	2753.32	1896.78	1359.34	1590.68
**Means**	#DIV/0!	#DIV/0!	#DIV/0!	#DIV/0!	#DIV/0!
**Sd**	#DIV/0!	#DIV/0!	#DIV/0!	#DIV/0!	#DIV/0!
**Median**	#NUM!	#NUM!	#NUM!	#NUM!	#NUM!

Post-60
2599.88
2030.95
3006.90
1900.21
2444.48
4280.48
1282.71
620.98
500.90
2070.82
642.45
1099.29
930.22
1712.98
2027.90
#DIV/0!
#DIV/0!
#NUM!

		HF POWER ms2		
**Suibjects**	**Baseline**	**Post-0**	**Post-15**	**Post-30**
1	421.85	1059.64	627.79	474.33
2	1092.56	1907.67	1253.73	1279.40
3	6153.15	5092.76	5360.89	3349.59
4	1204.84	2761.06	3290.81	2372.95
5	204.11	224.97	741.76	1221.88
6	693.30	891.09	658.41	843.58
7	1015.74	3530.89	2946.81	1734.03
8	2507.10	1151.21	1201.11	1174.41
9	658.91	834.84	755.29	381.80
10	445.67	4873.99	1404.93	983.36
11	824.40	146.98	236.99	856.86
12	320.13	3063.21	1114.60	366.09
13	82.25	481.41	1792.10	2059.55
14	488.70	3051.91	3136.41	2629.30
15	3188.18	2831.80	433.32	377.91
**Means**	#DIV/0!	#DIV/0!	#DIV/0!	#DIV/0!
**Sd**	#DIV/0!	#DIV/0!	#DIV/0!	#DIV/0!
**Median**	#NUM!	#NUM!	#NUM!	#NUM!

Post-45	Post-60
645.43	1229.55
2144.36	1056.52
3905.90	3059.66
2253.27	2279.25
1006.95	663.08
485.76	779.66
1734.03	2151.82
1741.31	784.84
971.71	135.84
1770.45	2183.70
140.70	196.98
485.54	606.68
3805.00	496.21
3737.06	1804.24
557.72	489.41
#DIV/0!	#DIV/0!
#DIV/0!	#DIV/0!
#NUM!	#NUM!

			LF\HF RATIC
Suibjects	Baseline	Post-0	Post-15
1	0.79	0.69	0.65
2	1.53	0.77	2.40
3	0.26	1.06	0.78
4	1.06	1.32	0.54
5	2.11	1.01	1.50
6	0.94	1.77	1.89
7	0.46	0.43	0.38
8	0.81	1.07	1.28
9	1.04	1.00	0.68
10	0.88	0.87	1.60
11	1.98	5.91	7.15
12	0.80	1.14	0.60
13	3.33	0.86	1.63
14	0.98	0.34	0.25
15	1.63	0.97	4.38
Means	#DIV/0!	#DIV/0!	#DIV/0!
Sd	#DIV/0!	#DIV/0!	#DIV/0!
Median	#NUM!	#NUM!	#NUM!

)		
**Post-30**	**Post-45**	**Post-60**
0.86	0.63	2.11
1.80	1.25	1.92
0.97	0.86	0.98
1.08	1.20	0.83
2.86	2.02	3.69
2.91	3.90	5.49
0.68	0.55	0.60
0.93	1.03	0.79
1.46	0.81	3.69
1.14	1.10	0.95
1.07	6.33	3.26
3.11	3.47	1.81
1.15	0.63	1.87
0.35	0.42	0.95
3.60	2.85	4.14
#DIV/0!	#DIV/0!	#DIV/0!
#DIV/0!	#DIV/0!	#DIV/0!
#NUM!	#NUM!	#NUM!

Systolic Blood Pressure mmHg						
**Suibjects**	**Baseline**	**Post-0**	**Post-15**	**Post-30**	**Post-45**	**Post-60**
1	117	127	117	118	123	121
2	108	114	107	114	114	109
3	109	110	105	110	106	106
4	118	107	107	101	101	99
5	103	110	112	115	101	103
6	121	119	117	109	114	114
7	108	113	112	111	111	118
8	115	99	103	100	100	109
9	116	104	99	99	120	107
10	114	115	89	97	98	107
11	105	108	107	109	110	110
12	111	108	106	105	108	105
13	103	109	110	108	103	115
14	101	104	99	103	98	91
15	105	105	106	108	107	105
**Means**	110,27	110,13	106,40	107,13	107,60	107,93
**Sd**	6,26	6,84	7,21	6,20	7,76	7,46
**Median**	109,00	109,00	107,00	108,00	107,00	107,00

Diastolic Blood Pressure mmHg					
**Suibjects**	**Baseline**	**Post-0**	**Post-15**	**Post-30**	**Post-45**
1	79	78	76	78	84
2	70	78	72	78	75
3	83	77	77	73	74
4	81	69	77	73	71
5	70	67	71	83	68
6	79	76	79	73	72
7	76	71	80	80	79
8	79	77	81	77	82
9	65	61	63	64	70
10	78	72	59	74	60
11	75	69	73	71	74
12	74	73	68	72	80
13	68	68	70	64	74
14	62	62	59	76	68
15	60	68	73	69	67
**Means**	73,27	71,07	71,87	73,67	73,20
**Sd**	7,11	5,50	7,08	5,35	6,34
**Median**	75,00	71,00	73,00	73,00	74,00

Post-60
80
82
79
73
63
76
80
80
67
70
79
78
74
71
71
74,87
5,58
76,00

			RMSSD ms	
**Suibjects**	**Baseline**	**Post-0**	**Post-15**	**Post-30**
1	19.11	37.19	49.86	41.07
2	81.70	113.35	120.37	96.12
3	41.88	19.69	24.97	46.83
4	92.76	104.22	74.52	60.53
5	28.61	39.61	31.65	52.26
6	29.55	50.03	67.99	46.88
7	41.19	44.47	79.85	72.83
8	102.42	58.01	65.97	49.30
9	65.91	79.44	71.10	88.89
10	59.61	80.76	97.92	88.86
11	49.98	121.64	77.57	59.05
12	14.15	95.60	98.24	84.92
13	16.83	30.18	65.66	64.21
14	35.39	81.22	67.62	52.26
15	71.12	94.47	88.68	88.33
**Means**	#DIV/0!	#DIV/0!	#DIV/0!	#DIV/0!
**Sd**	#DIV/0!	#DIV/0!	#DIV/0!	#DIV/0!
**Median**	#NUM!	#NUM!	#NUM!	#NUM!

**Post-45**	**Post-60**
49.96	36.47
85.61	66.90
19.41	25.95
75.76	70.15
44.21	54.08
44.95	40.47
86.47	66.38
63.49	49.37
109.80	62.48
35.00	32.67
95.96	96.44
82.45	75.92
90.52	33.91
44.21	54.08
97.68	92.79
#DIV/0!	#DIV/0!
#DIV/0!	#DIV/0!
#NUM!	#NUM!

			**SDNN ms**
**Suibjects**	**Baseline**	**Post-0**	**Post-15**
1	41.71	53.59	68.24
2	91.25	87.95	95.20
3	59.44	54.97	63.02
4	118.28	87.79	73.70
5	62.08	71.11	38.69
6	50.98	75.93	71.81
7	53.52	44.54	77.42
8	93.09	73.50	85.72
9	79.13	84.48	66.09
10	73.31	89.58	129.99
11	65.60	126.83	104.82
12	30.18	74.55	79.16
13	44.20	38.03	95.78
14	50.08	73.68	67.94
15	90.77	90.78	86.91
**Means**	#DIV/0!	#DIV/0!	#DIV/0!
**Sd**	#DIV/0!	#DIV/0!	#DIV/0!
**Median**	#NUM!	#NUM!	#NUM!

Post-30	Post-45	Post-60
72.93	83.13	64.91
87.66	87.60	119.59
74.01	62.08	71.88
79.53	74.95	95.68
58.66	52.62	62.75
76.04	70.41	62.50
110.07	81.55	78.38
61.79	87.96	52.30
105.68	144.66	64.81
126.58	47.29	99.00
76.30	98.53	101.01
91.51	76.10	62.02
86.04	87.71	78.51
58.66	52.62	62.75
96.41	95.91	106.89
#DIV/0!	#DIV/0!	#DIV/0!
#DIV/0!	#DIV/0!	#DIV/0!
#NUM!	#NUM!	#NUM!

		LN (F
**Suibjects**	**Baseline**	**Post-0**
1	2.95	3.62
2	4.40	4.73
3	3.73	2.98
4	4.53	4.65
5	3.35	3.68
6	3.39	3.91
7	3.72	3.79
8	4.63	4.06
9	4.19	4.37
10	4.09	4.39
11	3.91	4.80
12	2.65	4.56
13	2.82	3.41
14	3.57	4.40
15	4.26	4.55
**Means**	#DIV/0!	#DIV/0!
**Sd**	#DIV/0!	#DIV/0!
**Median**	#NUM!	#NUM!

ƦMSSD) ms			
**Post-15**	**Post-30**	**Post-45**	**Post-60**
3.91	3.72	3.91	3.60
4.79	4.57	4.45	4.20
3.22	3.85	2.97	3.26
4.31	4.10	4.33	4.25
3.43	3.96	3.79	3.99
4.22	3.85	3.81	3.70
4.38	4.29	4.46	4.20
4.19	3.90	4.15	3.90
4.26	4.49	4.70	4.13
4.58	4.49	3.56	3.49
4.35	4.08	4.56	4.57
4.59	4.44	4.41	4.33
4.18	4.16	4.51	3.52
4.21	3.96	3.79	3.99
4.49	4.48	4.58	4.53
#DIV/0!	#DIV/0!	#DIV/0!	#DIV/0!
#DIV/0!	#DIV/0!	#DIV/0!	#DIV/0!
#NUM!	#NUM!	#NUM!	#NUM!

**Suibjects**	**Baseline**
1	2
2	58
3	14
4	59
5	7
6	8
7	24
8	64
9	39
10	45
11	32
12	1
13	1
14	16
15	44
**Means**	27,60
**Sd**	22,63
**Median**	24,00

Suibjects	PNN50% Post-0	Post-15	Post-30	Post-45	Post-60
1	16	30	17	30	19
2	67	74	69	60	42
3	1	4	30	2	7
4	63	49	42	46	44
5	8	6	32	30	51
6	34	47	26	25	23
7	24	46	46	47	40
8	44	39	30	37	9
9	61	60	58	74	54
10	54	61	67	14	10
11	69	47	36	59	51
12	67	66	54	53	36
13	7	35	40	57	13
14	56	48	32	30	51
15	60	49	49	52	50
**Means**	42,07	44,07	41,87	41,07	33,33
**Sd**	24,75	19,57	15,18	19,35	17,80
**Median**	54,00	47,00	40,00	46,00	40,00

Mean R-R ms					
**Baseline**	**Post-0**	**Post-15**	**Post-30**	**Post-45**	**Post-60**
653.08	748.85	797.68	755.07	783.10	761.27
969.98	1022.83	1046.83	1013.67	1010.04	967.40
634.55	695.40	692.40	866.99	692.66	711.53
1014.32	1105.18	1045.11	1006.56	1044.81	1034.39
715.35	760.20	791.49	695.05	668.30	678.51
889.55	966.94	1041.06	969.57	946.29	946.75
852.58	774.94	827.53	798.02	849.94	804.10
813.00	935.09	897.00	880.06	926.73	833.73
870.51	932.22	901.96	954.63	1034.88	916.53
872.42	919.26	992.82	1030.73	808.91	782.15
830.15	947.65	730.80	689.00	927.65	893.49
677.43	932.25	945.60	852.14	918.14	936.86
641.17	678.85	763.80	787.90	880.27	733.98
674.02	769.45	732.94	695.05	668.30	678.51
861.59	919.70	916.76	945.23	962.80	938.31
#DIV/0!	#DIV/0!	#DIV/0!	#DIV/0!	#DIV/0!	#DIV/0!
#DIV/0!	#DIV/0!	#DIV/0!	#DIV/0!	#DIV/0!	#DIV/0!
#NUM!	#NUM!	#NUM!	#NUM!	#NUM!	#NUM!

TOTAL POWER ms2						
**Suibjects**	**Baseline**	**Post-0**	**Post-15**	**Post-30**	**Post-45**	**Post-60**
1	583.73	1678.15	2788.43	2567.34	4409.88	2090.02
2	3807.36	4700.59	5737.87	3717.03	3249.20	4564.24
3	2453.67	1015.29	1930.82	1772.19	1031.91	839.43
4	5436.46	5467.76	2742.23	3077.47	3334.23	3762.65
5	1117.80	1257.99	552.32	2938.91	712.99	2605.74
6	887.44	1880.41	3036.88	2385.52	2086.58	1577.37
7	1292.05	1252.99	4214.89	3870.08	4253.88	3948.25
8	4541.55	2381.00	2471.27	2268.83	3537.17	1405.81
9	3350.70	5798.54	3111.46	5233.73	7787.73	2021.52
10	2084.98	1949.68	4668.89	2886.33	923.15	2256.05
11	1968.19	12796.33	4493.90	3520.41	6739.67	7331.11
12	362.13	5054.06	4108.85	4948.31	2696.38	3434.52
13	356.45	893.91	4704.60	4429.94	6206.02	1426.43
14	1265.54	5000.29	3894.31	2938.91	712.99	2605.74
15	3983.54	4090.98	3013.79	3021.98	6697.04	6694.72
**Means**	#DIV/0!	#DIV/0!	#DIV/0!	#DIV/0!	#DIV/0!	#DIV/0!
**Sd**	#DIV/0!	#DIV/0!	#DIV/0!	#DIV/0!	#DIV/0!	#DIV/0!
**Median**	#NUM!	#NUM!	#NUM!	#NUM!	#NUM!	#NUM!

LF POWER ms2					
**Suibjects**	**Baseline**	**Post-0**	**Post-15**	**Post-30**	**Post-45**
1	369.30	1081.43	1786.98	1773.84	3089.31
2	2086.91	2037.55	2781.39	1645.05	1924.50
3	1629.27	868.31	1693.83	915.33	891.20
4	2722.38	244.11	940.54	1596.06	1616.18
5	867.19	821.70	260.74	969.32	972.89
6	642.30	727.51	1479.76	1554.75	1228.83
7	612.33	638.27	1606.92	2276.15	1797.38
8	2034.44	1229.79	1540.16	1094.41	1795.87
9	1775.93	3246.90	1330.75	2226,82	2188.72
10	1134.62	638.47	1853.16	928.48	337.94
11	927.42	4308.56	1201.32	1349.38	3108.27
12	464.22	1523.17	1131.69	2001.51	962.35
13	274.20	412.50	2912.51	2370.40	2401.02
14	294.36	1261.63	1133.12	969.32	972.89
15	2560.72	1838.50	1542.93	1486.25	3688.91
**Means**	#DIV/0!	#DIV/0!	#DIV/0!	2.226,82	#DIV/0!
**Sd**	#DIV/0!	#DIV/0!	#DIV/0!	#DIV/0!	#DIV/0!
**Median**	#NUM!	#NUM!	#NUM!	2.226,82	#NUM!

**Post-60**
1540.52
3537.61
642.45
2671.31
969.58
969.54
1909.35
620.98
587.92
1913.55
3292.48
1282.71
930.22
969.58
4776.06
#DIV/0!
#DIV/0!
#NUM!

		**HF POWER ms2**		
**Suibjects**	**Baseline**	**Post-0**	**Post-15**	**Post-30**
1	214.43	596.72	1001.44	793.50
2	1720.44	26.63.04	2956.48	2071.98
3	824.40	146.98	236.99	856.86
4	2714.08	3020.65	1801.69	1481.41
5	250.61	436.29	291.58	1968.99
6	245.14	1152.90	1557.12	830.77
7	679.72	614.72	2607.97	1593.94
8	2507.10	1151.21	1201.11	1174.41
9	1774.77	2551.65	1780.72	3006.91
10	950.36	1311.21	2815.73	1957.85
11	1040.77	8487.77	3292.58	2171.04
12	1015.74	3530.89	2946.81	1734.03
13	82.25	481.41	1792.10	2059.55
14	971.18	3738.67	2761.19	1968.99
15	1422.82	2252.48	1470.86	1535.73
**Means**	#DIV/0!	#DIV/0!	#DIV/0!	#DIV/0!
**Sd**	#DIV/0!	#DIV/0!	#DIV/0!	#DIV/0!
**Median**	#NUM!	#NUM!	#NUM!	#NUM!

Post-45	Post-60
1320.58	549.50
1324.70	1026.63
140.70	196.98
1718.05	1191.34
3805.00	496.21
857.74	607.83
2456.50	2038.90
1741.31	784.84
5599.02	1433.60
585.21	342.50
3631.41	4038.63
1734.03	2151.82
3805.00	496.21
1568.32	1636.16
3008.13	1918.66
#DIV/0!	#DIV/0!
#DIV/0!	#DIV/0!
#NUM!	#NUM!

LF\HF RATIC			
**Suibjects**	**Baseline**	**Post-0**	**Post-15**
1	1.72	1.81	1.78
2	1.21	0.77	0.94
3	1.98	5.91	7.15
4	1.00	0.81	0.52
5	3.46	1.88	0.89
6	2.62	0.63	0.95
7	0.90	1.04	0.62
8	0.81	1.07	1.28
9	1.00	1.27	0.75
10	1.19	0.49	0.66
11	0.89	0.51	0.36
12	0.46	0.43	0.38
13	3.33	0.86	1.63
14	0.30	0.34	0.41
15	1.80	0.82	1.05
**Means**	#DIV/0!	#DIV/0!	#DIV/0!
**Sd**	#DIV/0!	#DIV/0!	#DIV/0!
**Median**	#NUM!	#NUM!	#NUM!

)		
**Post-30**	**Post-45**	**Post-60**
2.24	2.34	2.80
0.79	1.45	3.45
1.07	6.33	3.26
1.08	0.94	2.16
0.49	0.63	1.87
1.87	1.43	1.60
1.43	0.73	0.94
0.93	1.03	0.79
0.74	0.39	0.41
0.47	0.58	5.59
0.62	0.86	0.82
0.68	0.55	0.60
1.15	0.63	1.87
0.49	0.62	0.59
0.97	1.23	2.49
#DIV/0!	#DIV/0!	#DIV/0!
#DIV/0!	#DIV/0!	#DIV/0!
#NUM!	#NUM!	#NUM!

Systolic Blood Pressure mmHg						
**Suibjects**	**Baseline**	**Post-0**	**Post-15**	**Post-30**	**Post-45**	**Post-60**
1	124	114	111	113	127	114
2	105	114	117	117	109	121
3	115	115	116	115	101	120
4	105	105	106	108	107	105
5	107	104	108	110	108	104
6	118	122	120	119	117	109
7	110	109	120	115	111	124
8	101	104	98	102	103	97
9	102	94	106	108	100	106
10	116	111	115	112	113	108
11	120	106	118	107	112	119
12	102	114	123	111	108	108
13	104	103	99	112	98	111
14	103	99	112	100	99	97
15	100	97	104	100	100	96
**Means**	108,80	107,40	111,53	109,93	107,53	109,27
**Sd**	7,80	7,68	7,79	5,85	7,90	8,97
**Median**	105,00	106,00	112,00	111,00	108,00	108,00

Diastolic Blood Pressure mmHg					
**Suibjects**	**Baseline**	**Post-0**	**Post-15**	**Post-30**	**Post-45**
1	78	72	75	75	95
2	69	72	76	79	79
3	80	69	77	67	65
4	60	68	73	69	67
5	66	66	72	64	66
6	72	74	72	76	74
7	73	74	79	81	75
8	67	65	67	67	71
9	68	66	66	68	63
10	72	74	72	76	68
11	66	79	75	75	79
12	71	79	80	71	69
13	65	67	70	69	70
14	74	68	69	70	61
15	70	71	70	72	72
**Means**	70,07	70,93	72,87	71,93	71,60
**Sd**	5,13	4,48	4,14	4,88	8,36
**Median**	70,00	71,00	72,00	71,00	70,00

Post-60
76
79
66
71
67
71
84
64
71
76
83
71
79
72
71
73,40
5,95
71,00

		RMSSD ms		
**Suibjects**	**Baseline**	**Post-0**	**Post-15**	**Post-30**
1	32.86	51.49	45.68	47.96
2	88.02	105.17	87.74	79.00
3	26.13	61.17	56.91	85.50
4	71.12	94.47	88.68	88.33
5	27.78	32.15	36.57	36.41
6	27.86	41.13	34.99	31.80
7	35.51	59.37	68.06	82.40
8	32.68	28.91	61.73	34.60
9	44.71	55.04	58.55	45.39
10	34.37	67.22	76.63	70.90
11	22.57	18.90	45.68	40.58
12	41.29	73.12	103.53	128.28
13	21.81	44.43	50.49	49.91
14	18.90	90.20	42.08	67.54
15	79.59	109.70	34.99	31.80
**Means**	#DIV/0!	#DIV/0!	#DIV/0!	#DIV/0!
**Sd**	#DIV/0!	#DIV/0!	#DIV/0!	#DIV/0!
**Median**	#NUM!	#NUM!	#NUM!	#NUM!

Post-45	Post-60
42.15	52.23
65.67	91.53
87.87	81.64
97.68	92.79
33.62	39.26
35.71	39.98
71.36	58.04
27.29	38.12
30.45	16.37
48.71	69.95
87.87	131.39
134.33	67.88
48.61	37.63
122.22	137.97
35.71	39.98
#DIV/0!	#DIV/0!
#DIV/0!	#DIV/0!
#NUM!	#NUM!

			**SDNN ms**
**Suibjects**	**Baseline**	**Post-0**	**Post-15**
1	48.45	58.65	96.73
2	102.23	104.51	86.43
3	37.17	70.50	68.59
4	90.77	90.78	86.91
5	40.81	24.92	43.43
6	53.93	56.92	69.79
7	45.25	51.67	96.05
8	47.19	49.07	95.22
9	67.41	66.42	65.78
10	39.46	84.40	104.81
11	57.70	37.61	96.73
12	43.22	62.64	94.65
13	33.21	54.34	58.90
14	35.00	86.50	70.44
15	130.15	105.92	69.79
**Means**	#DIV/0!	#DIV/0!	#DIV/0!
**Sd**	#DIV/0!	#DIV/0!	#DIV/0!
**Median**	#NUM!	#NUM!	#NUM!

Post-30	Post-45	Post-60
91.57	96.62	81.39
109.54	122.04	102.34
92.40	93.61	93.46
96.41	95.91	106.89
64.94	35.64	54.15
64.96	77.10	79.01
76.26	93.21	78.57
44.37	35.66	61.94
60.01	70.55	46.93
96.21	72.15	113.47
67.45	93.61	153.18
118.81	120.03	61.23
53.56	56.05	49.08
83.90	135.50	141.11
64.96	77.10	79.01
#DIV/0!	#DIV/0!	#DIV/0!
#DIV/0!	#DIV/0!	#DIV/0!
#NUM!	#NUM!	#NUM!

		LN ([illegible]
**Suibjects**	**Baseline**	**Post-0**
1	3.49	3.94
2	4.48	4.66
3	3.26	4.11
4	4.26	4.55
5	3.32	3.47
6	3.33	3.72
7	3.57	4.08
8	3.49	3.36
9	3.80	4.01
10	3.54	4.21
11	3.12	2.94
12	3.72	4.29
13	3.08	3.79
14	2.94	4.50
15	4.38	4.70
**Means**	#DIV/0!	#DIV/0!
**Sd**	#DIV/0!	#DIV/0!
**Median**	#NUM!	#NUM!

RMSSD) ms			
**Post-15**	**Post-30**	**Post-45**	**Post-60**
3.82	3.87	3.74	3.96
4.47	4.37	4.18	4.52
4.04	4.45	4.48	4.40
4.49	4.48	4.58	4.53
3.60	3.59	3.52	3.67
3.55	3.46	3.58	3.69
4.22	4.41	4.27	4.06
4.12	3.54	3.31	3.64
4.07	3.82	3.42	2.80
4.34	4.26	3.89	4.25
3.82	3.70	4.48	4.88
4.64	4.85	4.90	4.22
3.92	3.91	3.88	3.63
3.87	4.21	4.81	4.93
3.55	3.46	3.58	3.69
#DIV/0!	#DIV/0!	#DIV/0!	#DIV/0!
#DIV/0!	#DIV/0!	#DIV/0!	#DIV/0!
#NUM!	#NUM!	#NUM!	#NUM!

**Suibjects**	**Baseline**
1	9
2	61
3	4
4	44
5	4
6	7
7	14
8	9
9	28
10	10
11	4
12	21
13	3
14	2
15	45
**Means**	17,67
**Sd**	18,50
**Median**	9,00

PNN50%					
**Post-0**	**Post-15**	**Post-30**	**Post-45**	**Post-60**	**Suibjects**
27	22	22	24	33	1
66	59	45	38	54	2
28	32	46	51	48	3
60	49	49	52	50	4
7	15	11	11	18	5
21	14	9	15	21	6
48	45	45	44	35	7
7	47	14	1	18	8
41	41	22	9	0	9
42	63	49	29	44	10
1	22	22	51	67	11
48	61	70	70	48	12
26	32	30	29	15	13
63	19	40	69	78	14
67	14	9	15	21	15
36,80	35,67	32,20	33,87	36,67	**Means**
22,17	17,73	18,41	21,80	21,44	**Sd**
41,00	32,00	30,00	29,00	35,00	**Median**

Mean R-R ms					
Baseline	Post-0	Post-15	Post-30	Post-45	Post-60
747.08	832.28	785.25	819.25	721.87	805.60
900.97	977.75	981.94	953.68	866.94	900.51
620.07	691.98	691.95	748.67	771.00	759.05
861.59	919.70	916.76	945.23	962.80	938.31
792.69	867.01	868.47	881.14	927.04	921.18
864.39	910.27	859.38	829.21	844.07	913.03
764.71	873.33	840.76	895.70	817.81	783.97
759.66	797.52	836.72	835.22	820.10	828.15
857.01	886.12	890.60	853.64	644.60	568.43
877.46	893.97	1040.89	893.59	846.67	922.57
652.30	691.44	785.25	835.39	866.94	1009.30
878.74	949.29	965.02	1016.44	1020.18	955.31
704.68	813.91	840.23	798.51	803.92	804.70
587.28	758.19	644.97	702.48	988.95	1002.89
738.39	861.55	868.47	881.14	927.04	921.18
#DIV/0!	#DIV/0!	#DIV/0!	#DIV/0!	#DIV/0!	#DIV/0!
#DIV/0!	#DIV/0!	#DIV/0!	#DIV/0!	#DIV/0!	#DIV/0!
#NUM!	#NUM!	#NUM!	#NUM!	#NUM!	#NUM!

TOTAL POWER ms2					
**Suibjects**	**Baseline**	**Post-0**	**Post-15**	**Post-30**	**Post-45**
1	1343.97	2464.36	1902.52	3302.12	2381.87
2	4174.15	5642.76	3164.31	6094.11	4753.64
3	520.01	4307.13	2110.68	5380.96	5915.28
4	3983.54	4090.98	3013.79	3021.98	6697.04
5	944.18	339.73	646.16	988.83	657.77
6	926.50	1670.29	1670.29	1737.25	2148.39
7	1176.78	1909.81	4680.91	3068.31	5543.46
8	892.26	857.10	3313.21	1067.82	781.08
9	1385.80	2237.63	2368.78	2127.13	1561.66
10	562.09	4305.58	2546.14	5699.63	2320.10
11	681.02	482.24	1902.52	1493.84	5915.28
12	1186.57	2499.01	5308.95	10406.69	10252.28
13	556.75	764.67	3770.52	1486.32	1228.53
14	709.93	6126.91	2439.50	3236.94	14979.28
15	8371.30	5585.12	1670.29	1737.25	2148.39
**Means**	#DIV/0!	#DIV/0!	#DIV/0!	#DIV/0!	#DIV/0!
**Sd**	#DIV/0!	#DIV/0!	#DIV/0!	#DIV/0!	#DIV/0!
**Median**	#NUM!	#NUM!	#NUM!	#NUM!	#NUM!

LF POWER ms2					
Suibjects	Baseline	Post-0	Post-15	Post-30	Post-60
1	650.67	1573.27	1244.11	2458.54	5060.14
2	1403.67	2736.50	1715.69	3960.83	6904.52
3	271.16	2538.58	973.66	1986.84	6823.51
4	2560.72	1838.50	1542.93	1486.25	6694.72
5	599.82	56.62	199.89	563.30	1148.59
6	616.17	1029.15	1896.78	1359.34	2517.31
7	677.10	677.51	2683.11	1207.56	3242.34
8	510.31	430.32	1584.32	497.31	1582.44
9	544.98	1050.85	1121.40	1353.74	594.74
10	239.03	2994.09	1062.48	3069.19	4672.42
11	523.61	348.20	1244.11	928.54	12508.14
12	539.82	727.57	1402.23	4550.86	2669.37
13	344.14	240.25	2818.28	702.94	892.28
14	395.69	1425.95	803.31	863.10	16234.59
15	5183.12	2753.32	1896.78	1359.34	2517.31
**Means**	#DIV/0!	#DIV/0!	#DIV/0!	#DIV/0!	#DIV/0!
**Sd**	#DIV/0!	#DIV/0!	#DIV/0!	#DIV/0!	#DIV/0!
**Median**	#NUM!	#NUM!	#NUM!	#NUM!	#NUM!

Post-45	Post-60
1896.11	4280.48
3034.83	3644.11
2837.74	3116.10
3688.91	4776.06
314.07	697.80
1590.68	2027.90
2511.56	1482.18
504.04	657.15
1082.72	433.25
1640.78	3401.39
2837.74	6382.34
3000.64	1025.29
663.92	399.72
6513.78	9764.12
1590.68	2027.90
#DIV/0!	#DIV/0!
#DIV/0!	#DIV/0!
#NUM!	#NUM!

		**HF POW**
**Suibjects**	**Baseline**	**Post-0**
1	693.30	891.09
2	2770.48	2906.26
3	248.85	1768.55
4	1422.82	2252.48
5	344.36	283.11
6	310.34	641.14
7	499.68	1332.30
8	381.95	426.77
9	840.82	1.186.788
10	323.06	1311.49
11	157.41	134.04
12	646.75	1771.43
13	212.61	524.42
14	314.25	4700.97
15	3188.18	2831.80
**Means**	#DIV/0!	1.186.788,00
**Sd**	#DIV/0!	#DIV/0!
**Median**	#NUM!	1.186.788,00

VER ms2						
Post-15	Post-30	Post-45	Post-60		Suibjects	Baseline
658.41	843.58	485.76	779.66		1	0.94
1448.63	2133.28	1718.82	3260.42		2	0.51
1137.02	3394.12	3077.54	3707.40		3	1.09
1470.86	1535.73	3008.13	1918.66		4	1.80
446.27	425.53	343.70	450.79		5	1.74
433.32	377.91	557.72	489.41		6	1.99
1997.79	1860.75	3031.89	1760.17		7	1.36
1728.90	570.50	277,04	907.30		8	1.34
1247.38	773.39	478.94	161.49		9	0.65
1483.66	2630.44	679.32	1271.04		10	0.74
658.41	565.31	3077.54	6125.80		11	3.33
3906.71	5855.83	7251.64	1644.09		12	0.83
952.24	783.38	564.61	492.55		13	1.62
1636.19	2373.85	8465.50	9764.12		14	1.26
433.32	377.91	557.72	489.41		15	1.63
#DIV/0!	#DIV/0!	277,04	#DIV/0!		**Means**	#DIV/0!
#DIV/0!	#DIV/0!	#DIV/0!	#DIV/0!		**Sd**	#DIV/0!
#NUM!	#NUM!	277,04	#NUM!		**Median**	#NUM!

LF\HF RATIO				
**Post-0**	**Post-15**	**Post-30**	**Post-45**	**Post-60**
1.77	1.89	2.91	3.90	5.49
0.94	1.18	1.86	1.77	1.12
1.44	0.86	0.59	0.92	0.84
0.82	1.05	0.97	1.23	2.49
0.20	0.45	1.32	0.91	1.55
1.61	4.38	3.60	2.85	4.14
0.43	1.24	0.65	0.83	0.84
1.01	0.92	0.87	1,81	0.74
0.89	0.90	1.75	2.26	2.68
2.28	0.72	1.17	2.42	2.68
2.60	1.89	1.64	0.92	1.04
0.41	0.36	0.78	0.41	0.62
0.46	2.96	0.90	1.18	0.81
0.30	0.49	0.36	0.77	0.66
0.97	4.38	3.60	2.85	4.14
#DIV/0!	#DIV/0!	#DIV/0!	1,81	#DIV/0!
#DIV/0!	#DIV/0!	#DIV/0!	#DIV/0!	#DIV/0!
#NUM!	#NUM!	#NUM!	1,81	#NUM!

Systolic Blood Pressure mmHg						
**Suibjects**	**Baseline**	**Post-0**	**Post-15**	**Post-30**	**Post-45**	**Post-60**
1	114	113	120	112	104	109
2	105	110	109	105	113	108
3	114	108	107	109	103	111
4	111	106	97	95	98	106
5	119	112	120	108	109	104
6	134	131	124	118	120	119
7	116	105	106	107	107	106
8	110	108	115	109	108	107
9	114	105	109	104	111	109
10	107	104	105	111	108	104
11	104	116	119	111	118	113
12	104	115	115	110	106	108
13	100	100	107	104	112	113
14	101	98	102	108	109	105
15	100	97	104	100	100	96
**Means**	110,20	108,53	110,60	107,40	108,40	107,87
**Sd**	8,98	8,48	7,80	5,41	6,00	5,18
**Median**	110,00	108,00	109,00	108,00	108,00	108,00

Diastolic Blood Pressure mmHg					
**Suibjects**	**Baseline**	**Post-0**	**Post-15**	**Post-30**	**Post-45**
1	78	75	75	78	70
2	67	74	76	75	68
3	74	71	69	69	73
4	65	66	68	66	70
5	64	71	73	71	68
6	82	80	88	75	76
7	69	70	68	69	70
8	71	68	79	69	69
9	68	69	64	63	65
10	65	67	68	73	71
11	66	76	77	73	77
12	73	76	76	79	74
13	64	68	70	72	78
14	68	70	75	74	71
15	70	71	70	72	72
**Means**	69,60	71,47	73,07	71,87	71,47
**Sd**	5,26	3,94	5,93	4,26	3,60
**Median**	68,00	71,00	73,00	72,00	71,00

Post-60
75
76
72
68
58
76
73
71
70
69
75
78
79
70
71
72,07
5,12
72,00

	RMSSD ms			
Suibjects	Baseline	Post-0	Post-15	Post-30
1	55.99	51.97	47.63	40.97
2	61.87	57.11	63.66	70.90
3	36.52	81.30	118.03	64.71
4	71.97	99.46	58.30	67.54
5	6.61	30.71	17.09	28.32
6	28.01	32.08	39.44	34.46
7	30.09	99.06	108.87	111.43
8	22.09	31.95	52.61	39.93
9	37.79	61.80	41.52	37.16
10	91.10	68.01	71.20	79.56
11	49.23	78.93	111.79	112.66
12	25.15	41.03	40.36	64.83
13	38.05	54.77	64.26	64.02
14	48.62	51.53	49.11	59.76
15	79.59	109.70	34.99	31.80
**Means**	#DIV/0!	#DIV/0!	#DIV/0!	#DIV/0!
**Sd**	#DIV/0!	#DIV/0!	#DIV/0!	#DIV/0!
**Median**	#NUM!	#NUM!	#NUM!	#NUM!

SDNN ms

Suibjects	Baseline	Post-0	Post-15	Post-45	Post-60
1	72.06	47.43	84.99	52.63	73.97
2	71.70	67.39	85.67	48.71	69.95
3	49.51	79.70	110.36	86.17	63.53
4	87.57	108.94	70.56	49.91	27.78
5	16.65	64.39	18.34	38.52	42.33
6	41.67	51.87	68.70	31.84	32.29
7	30.50	81.44	89.96	90.78	86.02
8	42.03	60.21	56.51	51.25	49.12
9	61.59	61.97	54.56	43.55	41.55
10	98.98	69.65	60.62	65.15	78.67
11	54.03	74.96	98.47	109.19	111.04
12	37.15	49.13	59.27	59.91	44.65
13	47.10	64.85	77.78	55.11	69.70
14	57.97	77.91	68.85	74.59	56.78
15	130.15	105.92	69.79	35.71	39.98
**Means**	#DIV/0!	#DIV/0!	#DIV/0!	#DIV/0!	#DIV/0!
**Sd**	#DIV/0!	#DIV/0!	#DIV/0!	#DIV/0!	#DIV/0!
**Median**	#NUM!	#NUM!	#NUM!	#NUM!	#NUM!

**Post-30**	**Post-45**	**Post-60**
67.34	70.80	89.24
96.21	72.15	113.47
81.55	91.39	84.64
83.90	53.56	40.81
40.90	54.36	53.41
52.86	44.55	61.35
90.74	92.57	83.11
58.20	69.91	65.28
45.61	65.86	66.60
78.49	55.29	120.30
102.72	133.96	124.89
75.82	78.40	71.99
79.00	71.23	94.31
87.83	87.44	67.95
64.96	77.10	79.01
#DIV/0!	#DIV/0!	#DIV/0!
#DIV/0!	#DIV/0!	#DIV/0!
#NUM!	#NUM!	#NUM!

		**LN (F**
**Suibjects**	**Baseline**	**Post-0**
1	4.03	3.70
2	4.13	4.04
3	3.60	4.40
4	4.28	4.60
5	1.89	3.42
6	3.33	3.47
7	3.40	4.60
8	3.10	3.46
9	3.63	4.12
10	4.51	4.22
11	3.90	4.37
12	3.22	3.71
13	3.64	4.00
14	3.88	3.94
15	4.38	4.70
**Means**	#DIV/0!	#DIV/0!
**Sd**	#DIV/0!	#DIV/0!
**Median**	#NUM!	#NUM!

RMSSD) ms			
**Post-15**	**Post-30**	**Post-45**	**Post-60**
3.86	3.71	3.96	4.30
4.15	4.26	3.89	4.25
4.77	4.17	4.46	4.15
4.07	4.21	3.91	3.32
2.84	3.34	3.65	3.75
3.67	3.54	3.46	3.47
4.69	4.71	4.51	4.45
3.96	3.69	3.94	3.89
3.73	3.62	3.77	3.73
4.27	4.38	4.18	4.37
4.72	4.72	4.69	4.71
3.70	4.17	4.09	3.80
4.16	4.16	4.01	4.24
3.89	4.09	4.31	4.04
3.55	3.46	3.58	3.69
#DIV/0!	#DIV/0!	#DIV/0!	#DIV/0!
#DIV/0!	#DIV/0!	#DIV/0!	#DIV/0!
#NUM!	#NUM!	#NUM!	#NUM!

**Suibjects**	**Baseline**
1	38
2	45
3	14
4	48
5	0
6	5
7	7
8	2
9	15
10	53
11	31
12	4
13	17
14	25
15	45
**Means**	23,27
**Sd**	18,66
**Median**	17,00

PNN50%					
Post-0	Post-15	Post-30	Post-45	Post-60	Suibjects
19	29	20	34	43	1
37	46	49	29	44	2
45	70	31	50	28	3
59	32	40	30	4	4
5	0	5	9	10	5
11	19	14	10	11	6
66	67	73	62	61	7
11	40	21	35	34	8
34	25	17	21	17	9
55	62	63	55	58	10
47	64	63	64	65	11
14	19	31	35	20	12
24	34	37	30	40	13
27	22	30	42	32	14
67	14	9	15	21	15
34,73	36,20	33,53	34,73	32,53	**Means**
20,95	21,44	20,74	17,38	19,18	**Sd**
34,00	32,00	31,00	34,00	32,00	**Median**

Mean R-R ms					
**Baseline**	**Post-0**	**Post-15**	**Post-30**	**Post-45**	**Post-60**
810.68	821.56	741.91	805.02	860.01	855.64
826.28	829.80	914.48	893.59	846.67	922.57
658.48	734.22	798.04	699.76	761.63	691.42
870.37	942.20	825.09	702.48	798.51	792.69
580.10	696.78	774.44	760.90	791.54	804.98
888.17	848.26	874.38	862.09	880.91	873.51
772.23	932.85	982.22	944.73	900.58	900.21
749.27	833.27	861.39	827.93	872.25	866.62
806.36	731.31	853.52	829.93	831.62	805.27
862.57	938.80	939.52	989.72	1019.28	1021.80
779.00	864.18	961.39	1014.14	970.76	988.95
809.25	868.82	886.36	928.48	955.20	883.43
744.63	838.71	830.52	806.64	805.16	833.53
656.89	660.66	652.66	667.33	713.98	677.60
738.39	861.55	868.47	881.14	927.04	921.18
#DIV/0!	#DIV/0!	#DIV/0!	#DIV/0!	#DIV/0!	#DIV/0!
#DIV/0!	#DIV/0!	#DIV/0!	#DIV/0!	#DIV/0!	#DIV/0!
#NUM!	#NUM!	#NUM!	#NUM!	#NUM!	#NUM!

TOTAL POWER ms2						
**Suibjects**	**Baseline**	**Post-0**	**Post-15**	**Post-30**	**Post-45**	**Post-60**
1	2622.62	1545.08	1780.06	1781.78	2155.53	3137.53
2	2279.72	2174.97	4001.37	5699.63	2320.10	4672.42
3	1228.94	4752.62	8844.55	3822.87	5092.27	3214.98
4	3237.93	8582.09	2681.62	3236.94	1486.32	944.18
5	97.70	967.54	157.23	596.46	502.27	2261.57
6	689.49	1116.85	2978.52	1681.72	1216.37	1424.16
7	673.59	4633.18	5164.12	6037.89	5493.83	4644.29
8	536.48	813.73	2298.67	1386.97	2253.85	1776.61
9	2172.72	2160.07	1417.29	1121.26	2343.33	2298.53
10	8460.63	1612.62	2017.95	3323.93	1502.86	3087.02
11	1844.65	3573.21	6430.96	8461.82	11538.22	11225.62
12	929.70	1028.90	2339.49	5049.81	3575.75	2968.09
13	909.47	1964.36	2767.65	2989.90	2731.79	3422.85
14	2555.00	3084.02	2029.92	3624.92	4090.97	3163.98
15	8371.30	5585.12	1670.29	1737.25	2148.39	2517.31
**Means**	#DIV/0!	#DIV/0!	#DIV/0!	#DIV/0!	#DIV/0!	#DIV/0!
**Sd**	#DIV/0!	#DIV/0!	#DIV/0!	#DIV/0!	#DIV/0!	#DIV/0!
**Median**	#NUM!	#NUM!	#NUM!	#NUM!	#NUM!	#NUM!

LF POWER ms2					
**Suibjects**	**Baseline**	**Post-0**	**Post-15**	**Post-30**	**Post-45**
1	1286.62	884.01	389.01	783.00	1046.10
2	2309.81	717.48	2926.34	3069.19	1640.78
3	634.97	1347.94	2836.82	1701.95	1531.64
4	1229.15	4607.72	1305.81	863.10	702.94
5	64.81	558.86	44.78	229.58	1429.82
6	405.61	713.66	1771.06	1043.75	703.09
7	257.21	2050.24	1415.73	2013.96	2117.99
8	318.20	320.18	1008.80	542.02	1085.77
9	1393.81	834.64	624.53	606.78	1547.59
10	4899.42	736.83	550.07	1496.11	478.01
11	677.05	928.25	1751.32	3434.89	5363.43
12	648.32	403.98	1740.60	3323.16	2201.19
13	342.47	800.48	1421.92	1368.49	1479.18
14	844.55	699.46	657.67	916.56	1140
15	5183.12	2753.32	1896.78	1359.34	1590.68
**Means**	#DIV/0!	#DIV/0!	#DIV/0!	#DIV/0!	1.140,00
**Sd**	#DIV/0!	#DIV/0!	#DIV/0!	#DIV/0!	#DIV/0!
**Median**	#NUM!	#NUM!	#NUM!	#NUM!	1.140,00

**Post-60**
1099.97
3401.39
1927.56
599.82
1710.12
1054.18
1632.17
850.88
1321.02
1082.02
4760.26
2159.52
2047.81
1093.81
2027.90
#DIV/0!
#DIV/0!
#NUM!

		**HF POWER ms2**		
**Suibjects**	**Baseline**	**Post-0**	**Post-15**	**Post-30**
1	1336.14	1331.16	1391.04	998.78
2	1069.91	1457.49	1075.03	2630.44
3	593.97	3404.68	6007.72	2120.93
4	2008.77	3974.37	1375.81	2373.85
5	32.89	407.69	112.45	366.88
6	283.88	403.19	1207.46	637.97
7	416.38	2582.94	3748.39	4023.93
8	218.28	493.55	1289.87	844.95
9	778.91	1325.44	792.76	514.47
10	3561.21	875.79	1467.88	1827.83
11	1167.60	2644.96	4679.64	5026.92
12	281.38	624.92	598.89	1726.65
13	567.00	1163.88	1345.73	1621.41
14	1710.45	2384.56	1372.25	2708.36
15	3188.18	2831.80	433.32	377.91
**Means**	#DIV/0!	#DIV/0!	#DIV/0!	#DIV/0!
**Sd**	#DIV/0!	#DIV/0!	#DIV/0!	#DIV/0!
**Median**	#NUM!	#NUM!	#NUM!	#NUM!

**Post-45**	**Post-60**
1109.43	2037.56
679.32	1271.04
3560.63	1287.42
783.38	344.36
578.99	551.45
513.28	369.98
3375.84	3012.12
1168.09	925.72
795.74	977.51
1024.84	2004.73
6174.79	6465.36
1374.56	808.57
1252.61	1375.05
2950.48	2070.17
557.72	489.41
#DIV/0!	#DIV/0!
#DIV/0!	#DIV/0!
#NUM!	#NUM!

			**LF\HF RATIC**
**Suibjects**	**Baseline**	**Post-0**	**Post-15**
1	0.96	1.34	0.28
2	2.16	0.49	2.72
3	1.07	0.40	0.47
4	0.61	1.16	0.95
5	1.97	1.37	0.40
6	1.43	1.77	1.47
7	0.62	0.79	0.38
8	1.46	0.65	0.78
9	1.79	0.63	0.79
10	1.38	0.84	0.37
11	0.58	0.35	0.37
12	2.30	0.65	2.91
13	0.60	0.69	1.06
14	0.49	0.29	0.48
15	1.63	0.97	4.38
**Means**	#DIV/0!	#DIV/0!	#DIV/0!
**Sd**	#DIV/0!	#DIV/0!	#DIV/0!
**Median**	#NUM!	#NUM!	#NUM!

)		
**Post-30**	**Post-45**	**Post-60**
0.78	0.94	0.54
1.17	2.42	2.68
0.80	0.43	1.50
0.36	0.90	1.74
0.63	1.47	3.10
1.64	1.37	2.85
0.50	0.63	0.54
0.64	0.93	0.92
1.18	1.94	1.35
0.82	0.47	0.54
0.68	0.87	0.74
1.92	1.60	2.67
0.84	1.18	1.49
0.34	0.39	0.53
3.60	2.85	4.14
#DIV/0!	#DIV/0!	#DIV/0!
#DIV/0!	#DIV/0!	#DIV/0!
#NUM!	#NUM!	#NUM!
